# 16S rRNA sequencing-based evaluation of the protective effects of Hua-Zhuo-Jie-Du on rats with chronic atrophic gastritis

**DOI:** 10.1186/s12906-022-03542-z

**Published:** 2022-03-16

**Authors:** Pingping Zhou, Tianxiao Yang, Miaochan Xu, Yuejia Zhao, Pengpeng Shen, Yangang Wang

**Affiliations:** 1 Hebei University of Chinese Medicine, Shijiazhuang, 050091 Hebei China; 2 The First Affiliated Hospital of Hebei University of Chinese Medicine, Shijiazhuang, 050011 Hebei China; 3 Shijiazhuang Pingan Hospital Co., Ltd, Shijiazhuang, 050025 Hebei China; 4 Beijing University of Chinese Medicine Third Affiliated Hospital, Anwai Xiaoguan Street No. 51, Chaoyang District, Beijing, 100029 China

**Keywords:** Chronic atrophic gastritis, Hua-Zhuo-Jie-Du, Intestinal flora, 16S rRNA sequencing

## Abstract

**Background:**

Disturbance of the intestinal flora is a pathogenic factor for chronic atrophic gastritis (CAG). Hua-Zhuo-Jie-Du (HZJD) has been shown to be an effective Chinese herbal preparation for treating CAG. However, the effects of HZJD on the intestinal flora of CAG is unclear. In this study, we probed the regulating effects of HZJD on intestinal microbes in CAG rats using 16S rRNA gene sequencing.

**Methods:**

High-performance liquid chromatography (HPLC) analysis was used to perform quality control of HZJD preparations. We then administered 1-methyl-3-nitro-1-nitrosoguanidine (200 μg/ml) to Sprague–Dawley rats to establish a CAG model. HZJD and vitacoenzyme were administered orally to these rats over a 10 week period. Hematoxylin and eosin (H&E) staining was performed to observe the histopathology of CAG rats. A rarefaction curve, species accumulation curve, Chao1 index, and ACE index were calculated to assess the alpha diversity. Principal component analysis (PCA), non-metric multi-dimensional scaling (NMDS), and unweighted pair group method with arithmetic mean (UPGMA) were conducted to examine the beta diversity. The LEfSe method was used to identify differential bacteria. Differential function analysis used PCA based on KEGG function prediction.

**Results:**

HPLC showed that our HZJD preparation method was feasible. H&E staining showed that HZJD significantly improved the pathological state of the gastric mucosa in CAG rats. The rarefaction curve and species accumulation curve showed that the sequencing data were reasonable. The Chao1 and ACE indices were significantly increased in CAG rats compared to the N group. Following HZJD and vitacoenzyme treatment, the Chao1 and ACE indices were decreased. PCA, NMDS, and UPGMA results showed that the M group was separated from the N, HZJD, and V groups, and LEfSe results showed that the relative abundance of *Akkermansia*, *Oscillospira*, *Prevotella*, and *CF231* were significantly higher in the N group. *Proteobacteria* and *Escherichia* were significantly enriched in the M group, *Allobaculum*, *Bacteroides*, *Jeotgalicoccus*, *Corynebacterium*, and *Sporosarcina* were significantly enriched in the V group, and *Firmicutes*, *Lactobacillus*, and *Turicibacter* were significantly enriched in the HZJD group.

**Conclusion:**

HZJD exhibited a therapeutic effect on the intestinal flora of CAG rats.

**Supplementary Information:**

The online version contains supplementary material available at 10.1186/s12906-022-03542-z.

## Background

Gastric carcinoma (GC) is a serious global health problem. With the prevalence of GC rising, this disease is currently the fifth most common cancer and the third leading cause of cancer death worldwide [1, 2]. Chronic atrophic gastritis (CAG) is defined as the occurrence of crucial transitional lesions from the normal gastric mucosa to GC. Its pathological features include decreased gastric acid secretion, thinning of the gastric mucosa, and decrease in proper gland morphology, with or without intestinal metaplasia or gastric epithelial dysplasia. CAG's main clinical manifestations include upper abdominal pain, bloating, and loss of appetite, which is occasionally accompanied by weight loss, diarrhea, or secondary anemia. CAG has become an important area of research given that suppression or reversion of CAG may serve to lower the incidence of GC [3, 4]. Emerging studies have demonstrated that the gut microbiome contributes to the regulation of gastrointestinal function and participates in various physiological and pathological processes, including inflammation and cell proliferation. Changes in intestinal flora promote the progression from CAG to GC [5–7].

Currently, therapies such as folic acid and vitamin B (12), endoscopic mucosal resection, and anti-H*p* treatment, are used in CAG treatment [8–11]. However, these treatments are often limited in clinical application and may have adverse effects. In China, many herbs based on traditional Chinese medicine (TCM) have been utilized to protect the gastric mucosa and reverse CAG [12, 13]. Hua-Zhuo-Jie-Du (HZJD) is a Chinese formula that is widely used for treating CAG that is based on the TCM theories of detoxicating and resolving dampness. HZJD consists of 11 herbs, including *Artemisia capillaris* Thunb. (Yinchen), *Scutellaria baicalensis* Georgi (Huangqin), *Coptis chinensis* Franch. (Huanglian), *Scutellaria barbata* D. Don (Banzhilian), *Scleromitrion diffusum* (Willd.) R. J. Wang (Baihuasheshecao), *Isatis tinctoria* L. (Banlangen), *Pogostemon cablin* (Blanco) Benth. (Guanghuoxiang), *Lobelia chinensis* Four. (Banbianlian), *Sophora flavescens* Aiton (Kushen), *Eupatorium fortunei* Turcz. (Peilan), and *Gynostemma pentaphyllum* (Thunb.) Makino (Jiaogulan). In previous research, through network pharmacology and experimental verification, the important components and possible targets of HZJD in the treatment of CAG were obtained [14]. Previous clinical experiments have also confirmed that HZJD had positive clinical effects [15]. In a recent study, it was shown that HZJD could inhibit the proliferation of precancerous gastric lesions and promote cell apoptos is by down-regulating lncRNA-517368 [16].

The effect of HZJD, however, on the intestinal flora in patients with CAG remains unclear. Therefore, in the current study, we sought to investigate whether HZJD inhibited the development of CAG by regulating the intestinal flora of CAG model rats.

## Materials and methods

### Experimental procedure

**Fig. 1:**
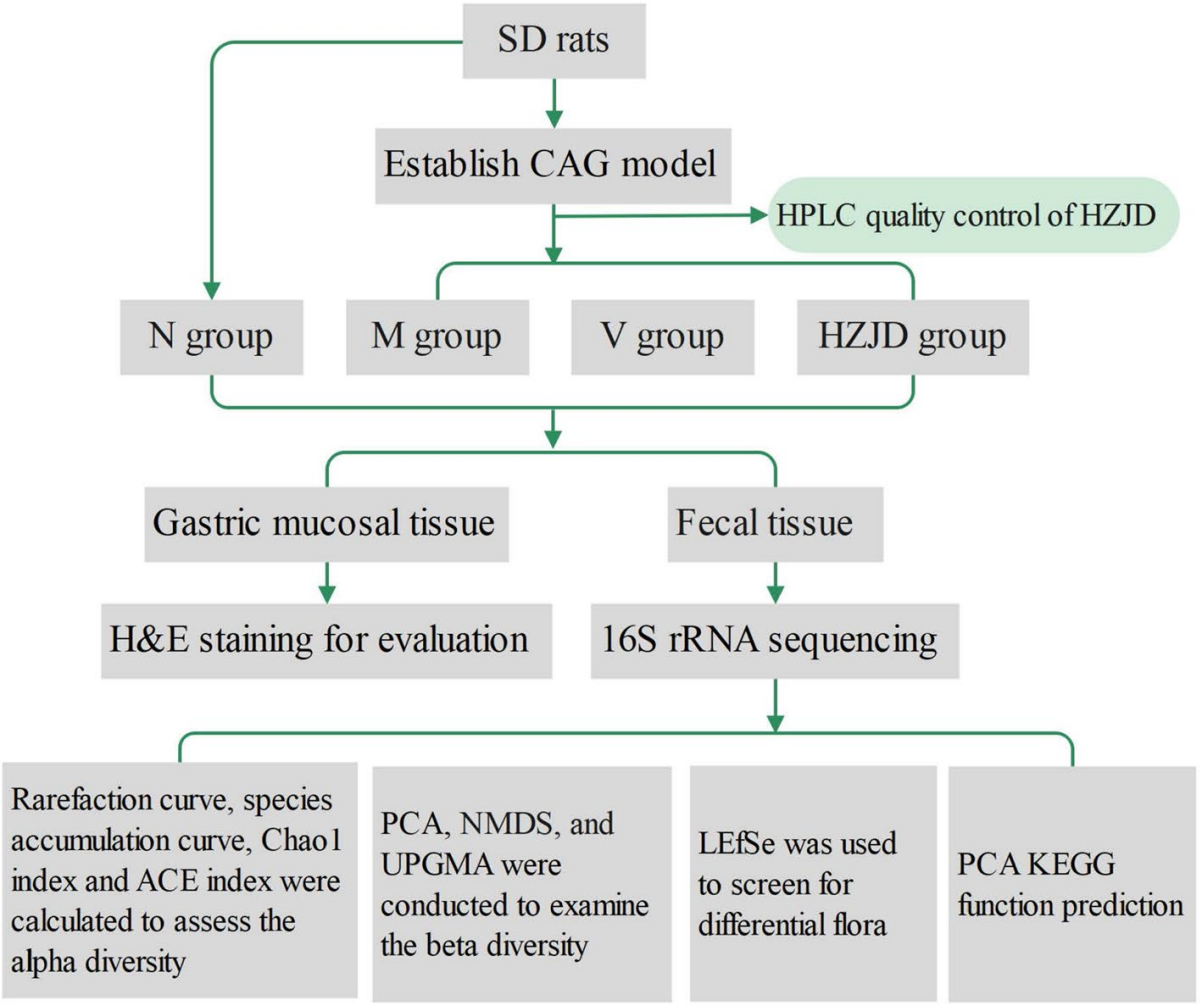
Experimental flow chart

An experimental flowchart for isolation of HZJD components is shown in Fig. 1.

### Drugs and reagents

1-Methyl-3-nitro-1-nitrosoguanidine (MNNG) was purchased from Tokyo Chemical Industry, Japan (Lot. NH8JH-BM). Vitacoenzyme tablets were purchased from LEPUHENGJIUYUAN Co., Ltd., China (No. 20190803). Sodium salicylate was purchased from Sangon Biotech, China (Lot. EC11BA0022). The following formula granules were provided by Guangdong Yifang Pharmaceutical Co., Ltd.: *Artemisia capillaris* Thunb. (Yinchen), 15 g; *Scutellaria baicalensis* Georgi (Huangqin), 12 g; *Coptis chinensis* Franch.(Huanglian), 12 g; *Scutellaria barbata* D. Don (Banzhilian), 15 g; *Scleromitrion diffusum* (Willd.) R. J. Wang (Baihuasheshecao), 15 g; *Isatis tinctoria* L.(Banlangen), 15 g; *Pogostemon cablin* (Blanco) Benth.(Guanghuoxiang), 9 g; *Lobelia chinensis* Lour. (Banbianlian), 15 g; *Sophora flavescens* Aiton (Kushen), 10 g; *Eupatorium fortunei* Turcz.(Peilan), 9 g; and *Gynostemma pentaphyllum* (Thunb.) Makino (Jiaogulan), 15 g.

### High-performance liquid chromatography (HPLC) analysis of HZJD

The quality and chemical constituents of our HZJD preparation were examined by HPLC. The condition optimization of the chemical fingerprint was as follows. An Agilent Eclipse XDB-C18 (5 μm, 4.6 × 250 mm), with methanol (A) and 0.1% phosphoric acid (B) as the mobile phase was used under full wavelength detection. Gradient elution was used for these solvents. For HPLC analysis, a 10 μl sample was injected into the column, with a column temperature of 35 °C. The detection was performed at 330 nm.

### Animals

The animal care and experimental procedures performed in this study were approved by the Institutional Animal Care and Use Committee of the Hebei College of Traditional Chinese Medicine (DWLL2019012). Seventy male Sprague–Dawley (SD) rats (body weight 150 – 180 g) were supplied by Liaoning Changsheng Biotechnology Co., Ltd. The rats were raised in a temperature controlled environment (24 °C ± 4 °C) under a 12/12 h light–dark cycle, with free access to water and food. Following a week of adaptation, the rats were randomly divided into a normal group (N, *n* = 8) and a CAG model group (*n* = 62). The N group had free access to clean water and normal diet. This CAG rat model was generated by administration of MNNG (200 μg/ml) combined with irregular fasting (1 day fasting and 1 day feeding), and 2% sodium salicylate was intragastrically administered once per fasting day on an empty stomach, as described previously [12, 13, 17].

Thereafter, at the 12^th^, 16^th^, 20^th^, and 24^th^ weeks, two rats from the model group were selected randomly and euthanized by intraperitoneal injection of 2% sodium pentobarbital (140 mg/kg). The stomach was removed for pathological examination to confirm successful establishment of a CAG model. Finally, the rats in the CAG model group were randomly divided into a model group (M, *n* = 8), a vitacoenzyme group (V, *n* = 8), and a HZJD group (HZJD, *n* = 8). The rats in the HZJD and V groups were treated with a daily dose of 14.81 g/kg/d HZJD and 0.08 g/kg/d vitacoenzyme. All animals were treated for 10 weeks, consecutively.

### Sample collection

Fresh feces were collected before pathological examination. Following fasting but were allowed to drink water for 24 h, the SD rats were euthanized with 2% sodium pentobarbital (140 mg/kg i.p.) and stomach samples were collected immediately. The gastric tissues and feces were stored at − 80 °C until future use.

### Pathological examination

Stomach samples were collected and then immersed in paraformaldehyde. Following tissue dehydration, the samples were embedded in paraffin and subsequently cut into 4-μm-thick sections. Hematoxylin–eosin (H&E) staining was performed for histological evaluation by light microscopy.

### Extraction of genomic DNA

The CTAB/SDS method was used to extract genomic DNA from gastric mucosal tissues. The DNA concentration and purity were monitored on a 1% agarose gel. The DNA was then diluted to 1 ng/μl with sterile water.

### Amplicon generation

Primers 16S V3-V4: 341F (5′-CCTAYGGGRBGCASCAG-3′) and 806R (5′-GGACTACNNGGGTATCTAAT-3′) were used for amplicon generation. The 16S rRNA gene was amplified using specific primers with barcodes. All PCR reactions were performed in a 30 μL reaction volume, with 15 μL Phusion® high-fidelity PCR Master Mix (New England Biolabs), 0.2 μM forward and reverse primers, and approximately 10 ng of template DNA. The thermal cycle included an initial denaturation at 98 °C for 1 min, followed by a cycle of denaturation at 98 °C for 30 s, annealing at 50 °C for 30 s, and extension at 72 °C for 60 s. Finally at 72 °C for 5 min.

### Quantification and qualification of PCR products

An equal volume of 1X loading buffer (containing SYB green) was mixed with PCR products, and electrophoresis on a 2% agarose gel was performed for detection. We selected samples with bright main bars between 400 and 450 bp for further experiments.

### Mixing and purification of PCR products

PCR products were mixed at an equivalent ratio before purifying with an AxyPrepDNA Gel Extraction Kit (AXYGEN).

### Library preparation and sequencing

Following the manufacturer’s recommendations, a sequencing library was generated using the NEB Next® Ultra™ DNA Library Preparation Kit for Illumina (NEB, USA), and an index sequence was added. The library quality was evaluated using a Qubit@2.0 Fluorometer (Thermo Scientific) and an Agilent Bioanalyzer 2100 system. Finally, the library was sequenced on the Illumina MiSeq/HiSeq 2500 platform, and 250 bp/300 bp paired end reads were generated.

### Data analysis

The raw data obtained by sequencing were spliced, filtered, and de-chimerized to obtain clean data to improve the accuracy and reliability of our results. We next divided the filtered sequences with ≥ 97% similarity into the same operational taxonomic unit (OTU). A rarefaction curve, species accumulation curve, Chao1 index, and ACE index were calculated to assess the alpha diversity of the species present in each sample and the depth of sequencing between groups. Based on this taxonomic information, a statistical analysis of community structure was conducted at each taxonomic level (phylum, class, order, family, genus, and species). Beta diversity was used to investigate the similarity of the bacterial community structures among the groups. Principal component analysis (PCA) and non-metric multi-dimensional scaling analysis (NMDS) were used to show beta diversity. The LEfSe uses linear discriminant analysis (LDA) to screen for differential flora. Finally, differential function analysis used PCA based on KEGG function prediction.

## Results

### HPLC profile for HZJD

**Fig. 2:**
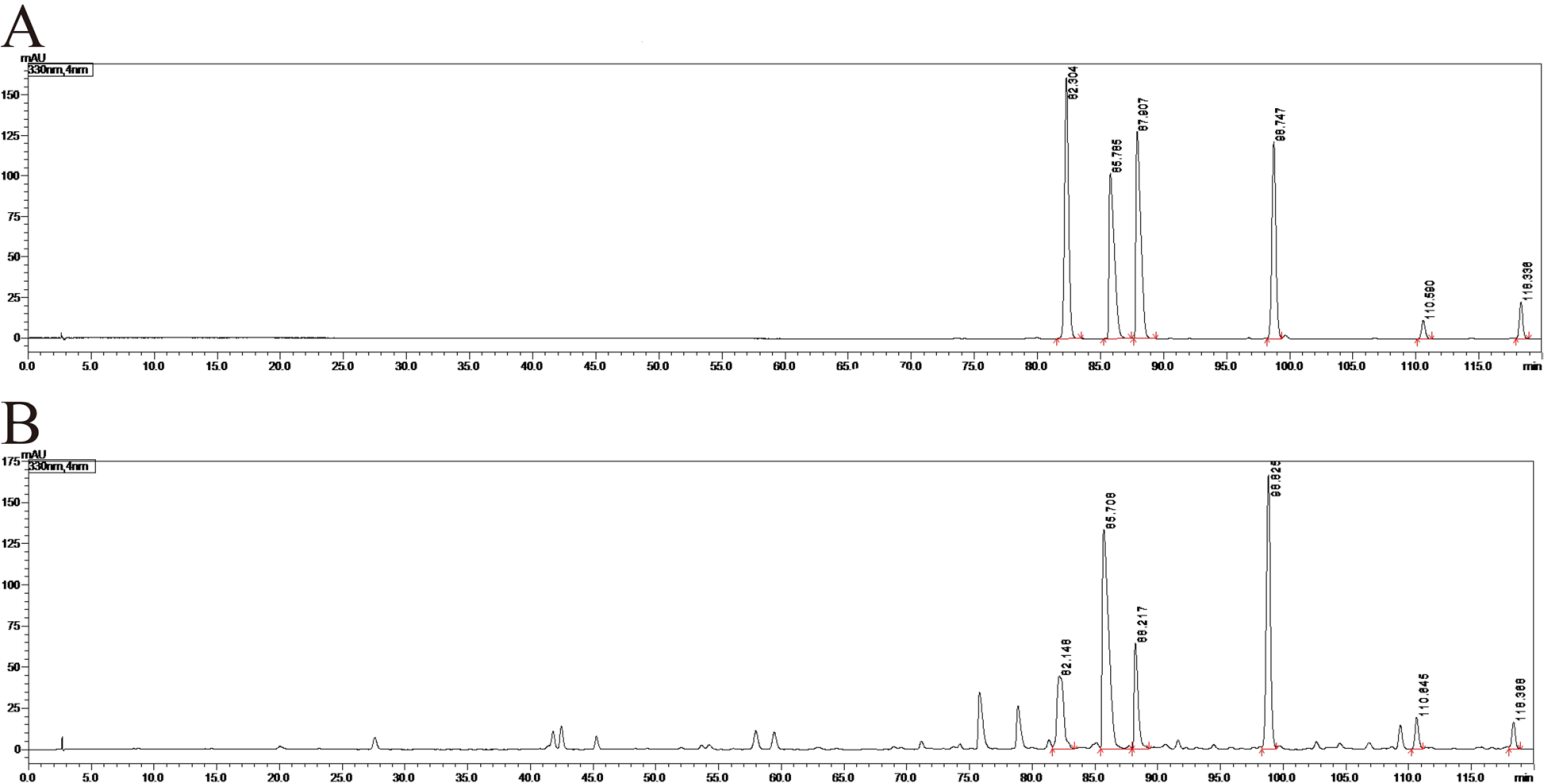
High-performance liquid chromatography (HPLC) chromatogram of a HZJD reference sample (**A**) and a test sample (**B**). From left to right, the index components are scutellarin, berberine, palmatine, baicalin, wogonin, and baicalein

Our previous clinical trials and animal experiments revealed that HZJD had a good inhibitory effect on CAG. We also examined the main components of HZJD granules to ensure the quality of their preparation. Fig. 2 shows the HPLC chromatograms of an HZJD reference sample (A) and of a test sample (B). The digital identification of the chromatographic peak on the picture is its retention time. From left to right, the index components are scutellarin, berberine, palmatine, baicalin, wogonin, and baicalein. The results showed that our preparation method for HZJD was both stable and feasible, which provides a reference for its quality control.

### Morphological changes of the gastric mucosa

**Fig. 3:**
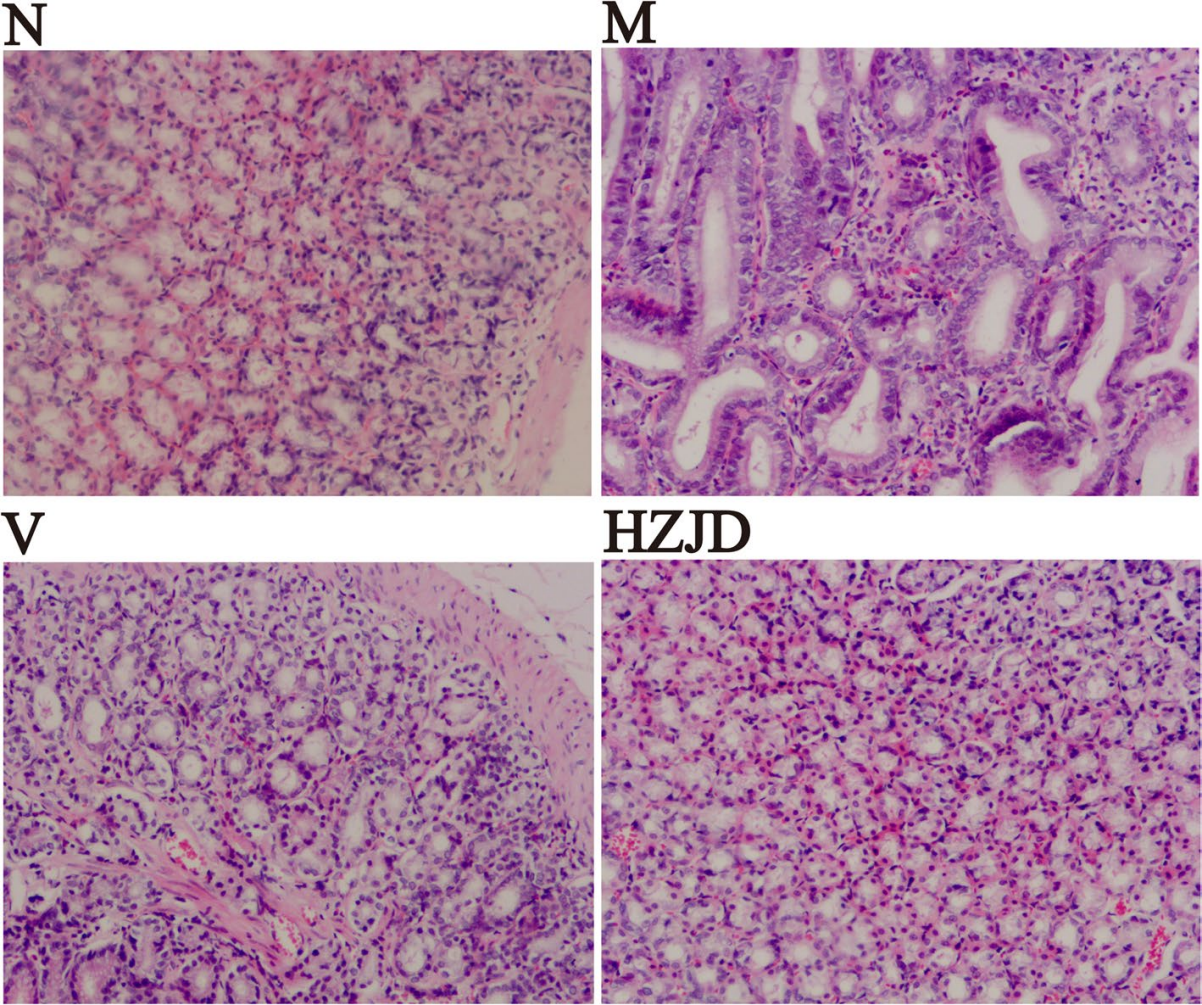
Representative images of the gastric mucosa of rats in different groups. (H&E × 100)

Figure 3 shows the histopathological changes of the gastric mucosa. H&E staining showed that in the N group rats, the gastric mucosal epithelial cells and the glands had preserved integrity and were arranged regularly. No expansion or hyperemia was observed. Additionally, no inflammatory cellular infiltration was observed in the submucosa and muscularis. In contrast, H&E staining showed that in the M group rats, the gastric glands were reduced and disordered, and the gastric mucosa layer was significantly reduced, which was accompanied by inflammatory cellular infiltration. These results suggested that a CAG rat model had been successfully established. Compared to the M group, H&E staining of V group rats showed that the arrangements of gastric glands were irregular, the structures of gastric glands were lacking, and the base layer of the mucosa was thicker, with no obvious improvement. However, the gastric mucosa morphology was significantly improved in the HZJD group compared to that in the M group. H&E staining indicated that the glands were neatly arranged and the complete glandular tube structure was clearly observable. No abnormal thickening of the muscularis mucosa was noted. Based on these pathological manifestations, HZJD was considered to be an effective treatment for CAG.

### Sequencing data preprocessing and quality control

A total of 2,881,791 raw sequences were obtained after 16S rRNA sequencing of the four groups of rats. After splicing, filtering, and de-chimerizing the raw data, 2,030,883 clean data sequences were obtained, with each sample containing 56,821 to 68,687 sequences (S1).

### Venn diagram

**Fig. 4:**
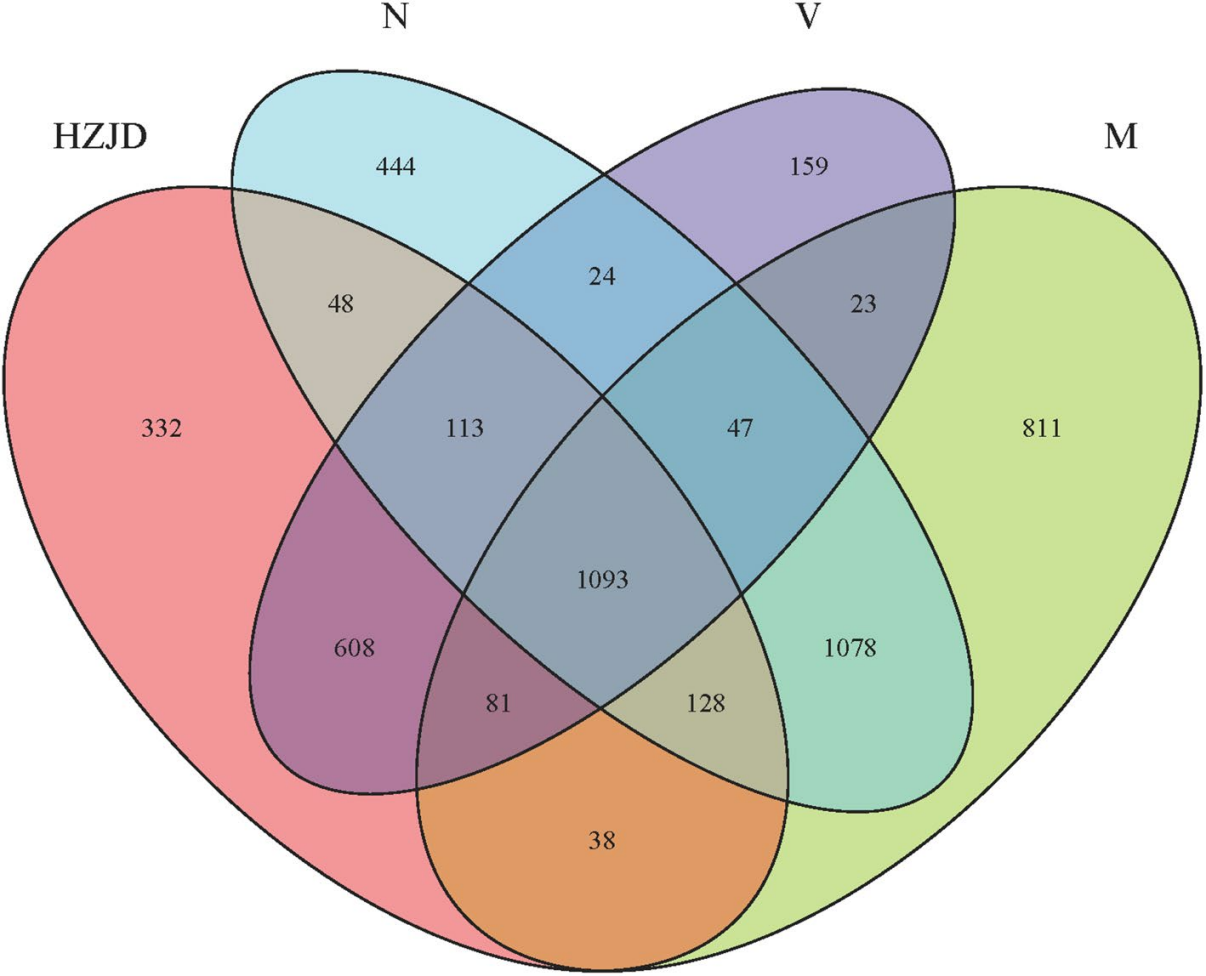
Venn diagram. Different colors represent different groups. The overlapping portions represent the common taxa between groups, and the non-overlapping portions represent unique taxa in each group

A Venn diagram was generated to define the core microbiome detected in most samples at the species level. We identified 2,975, 3,299, 2,441, and 2,148 species in the N, M, HZJD, and V groups, respectively (Fig. 4). Among these, 1,093 species were uniform and the other species were not shared in all groups; 444 species were detected in the N group only, whereas 811 species were detected in the M group only. Similarly, 332 species were found in the HZJD group only, and 159 species were found in the V group only.

### Alpha diversity

**Fig. 5:**
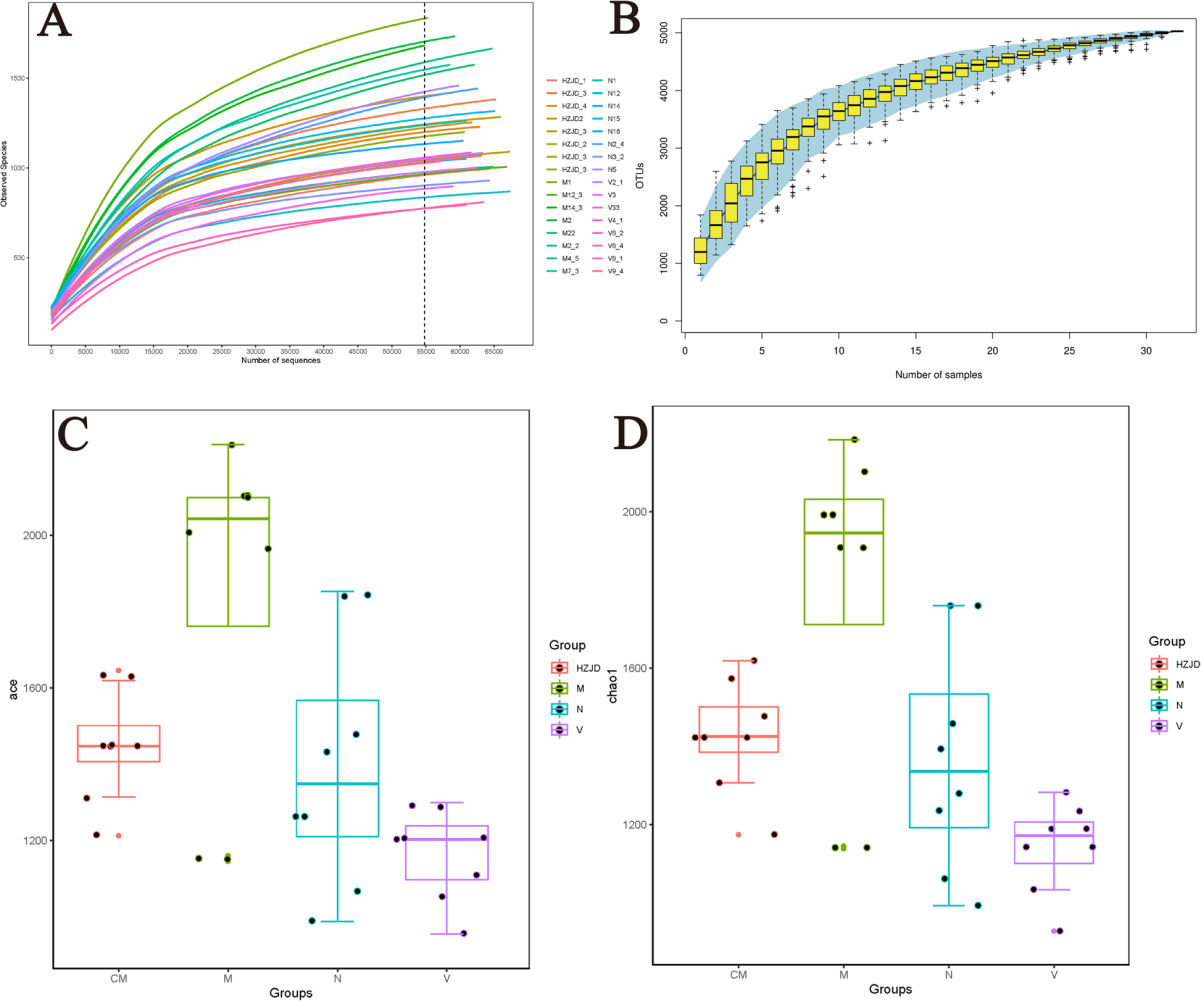
Alpha diversity indices from different groups. **A** Rarefaction curve. **B** Species accumulation curve. **C** Chao1 result. **D** ACE result. Different colors represent different groups

A fixed amount of sequencing data from each sample was extracted randomly and used to construct a rarefaction curve with the corresponding number of species. A rarefaction curve reflects the rationality of the amount of sequencing data. When the curve tends to be flat, it indicates that the amount of sequencing data is reasonable. As shown in Fig. 5A, the rarefaction curve gradually became flat, indicating that the sequencing data were reasonable in this study. A species accumulation curve can be used to fully determine whether a sample size is sufficient. A sharp rise in the position of the box chart indicates that the sample size is insufficient and the sample size needs to be increased, while the opposite indicates that the sample is sufficient and data analysis can be conducted. As shown in Fig. 5B, as the sample size increased, the box plot tended to be flat, indicating that the sample size was sufficient. We also calculated the Chao1 and ACE indices to assess the microbial diversity. Gut microbiota from the CAG rats demonstrated that the Chao1 and ACE indices were significantly increased compared to the N group. Following HZJD and vitacoenzyme treatment, the Chao1 and ACE indices were significantly decreased compared to the M group (Fig. 5C and D).

### Beta diversity

**Fig. 6:**
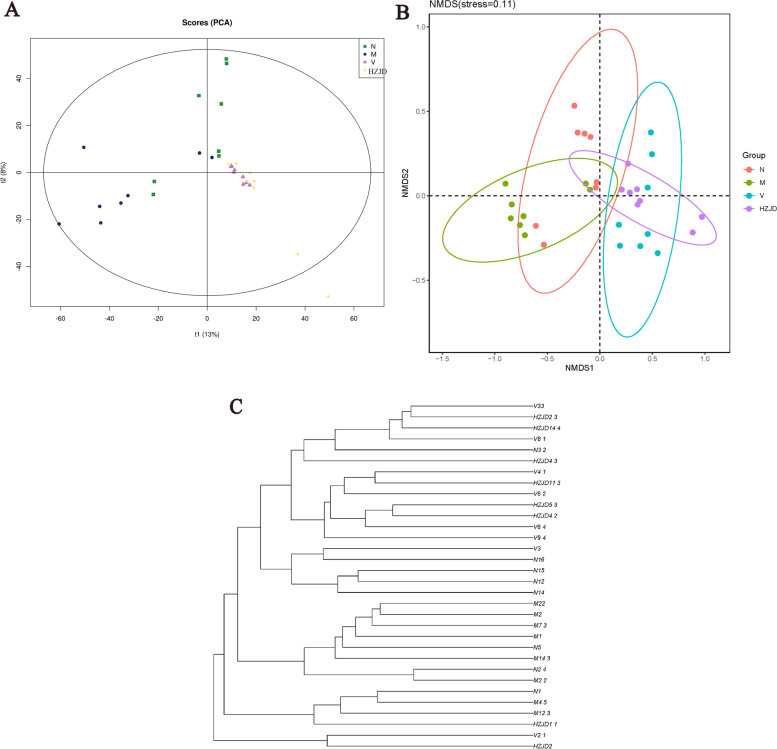
Beta diversity. **A** PCA score plot. **B** NMDS result. **C** UPGMA result

PCA can be used to show the similarity or difference in community composition between samples. Samples with high community structure similarity cluster together, while samples with large community structure differences become separated. The PCA score revealed that the community composition of the M group was far from that of the HZJD and V groups (Fig. 6A). The samples of the N, HZJD, and V groups were distributed on the right side of the t1 axis, which explained 13% of the total variations, indicating an influence of the HZJD constituents on the gut microbial composition. NMDS analysis is based on the species information contained in the sample in the form of points reflected in the multi-dimensional space, and the degree of difference between different samples is reflected by the distance between points, which can reflect the differences between groups and within groups of samples. The NMDS results also showed a separation between the HZJD and M groups (Fig. 6B). Moreover, to compare the similarity between different samples, the unweighted pair group method with arithmetic mean (UPGMA) was used. The results demonstrated that the majority of the gut microbes were clustered in their own groups, which was consistent with our PCA and NMDS results (Fig. 6C).

### Analysis of horizontal community structures across groups

**Fig. 7:**
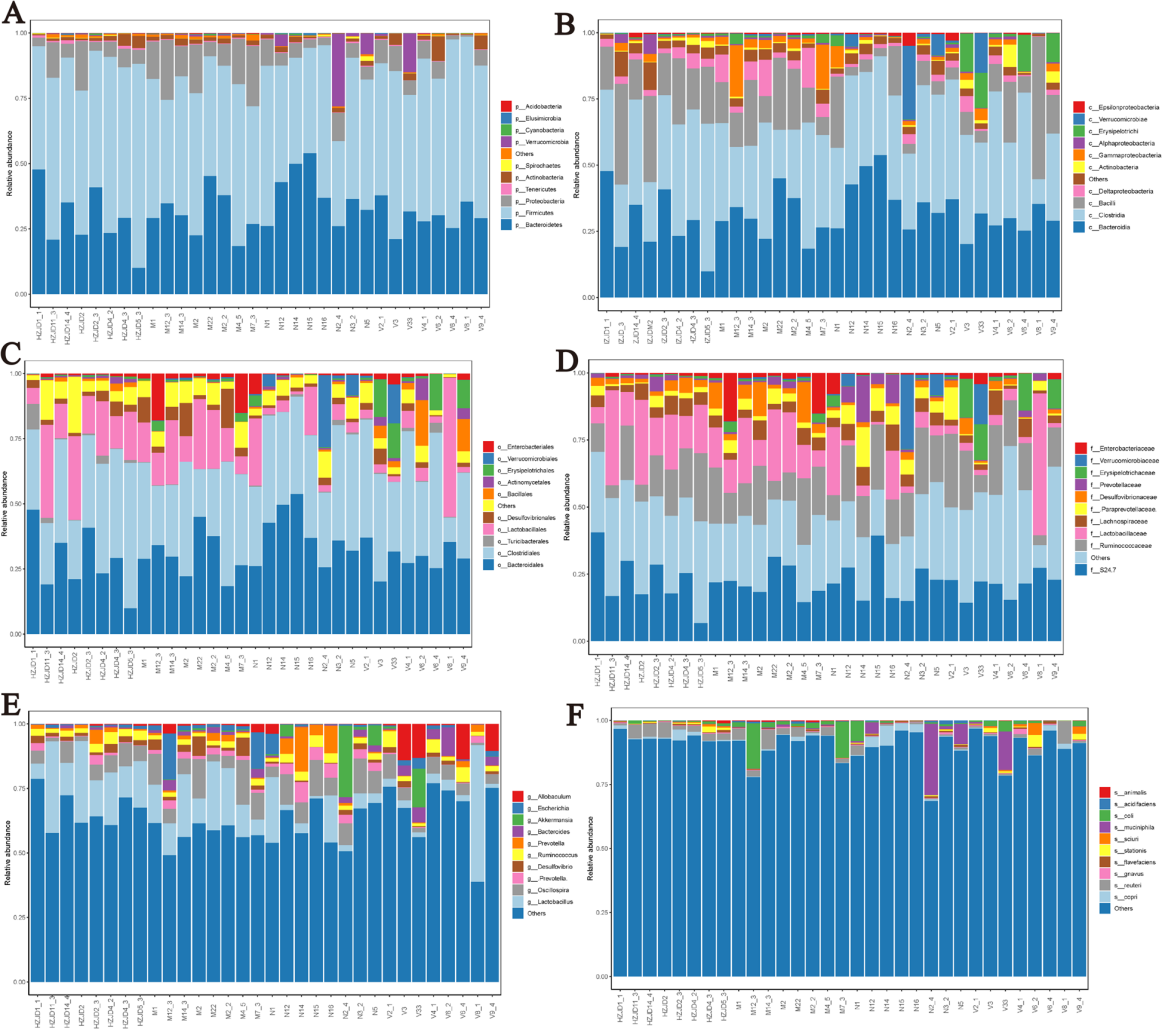
The top 10 abundant species at different levels **A** Phylum. **B** Class. **C** Order. **D** Family. **E** Genus. **F** Species

The top 10 abundant species among the 4 groups of rats were selected based on the results of species annotations, and a columnar cumulative chart of species relative abundance was generated to visually display the species with higher relative abundance and their proportions. At the phylum level, the top 10 abundant species were *Firmicutes*, *Bacteroidetes*, *Proteobacteria*, *Actinobacteria*, *Verrucomicrobia*, *Tenericutes*, *Spirochetes*, *Cyanobacteria*, *Elusimicrobia*, and *Acidobacteria* (Fig. 7A). At the genus level, the top 10 abundant species were *Lactobacillus, Oscillospira, Ruminococcus, Allobaculum, Prevotella, Escherichia, [Prevotella], Akkermansia, Desulfovibrio,* and *Bacteroides* (Fig. 7E)*.* The top 10 species of the other levels are shown in Fig. 7B, C, D and F.

### LEfSe analysis

**Fig. 8:**
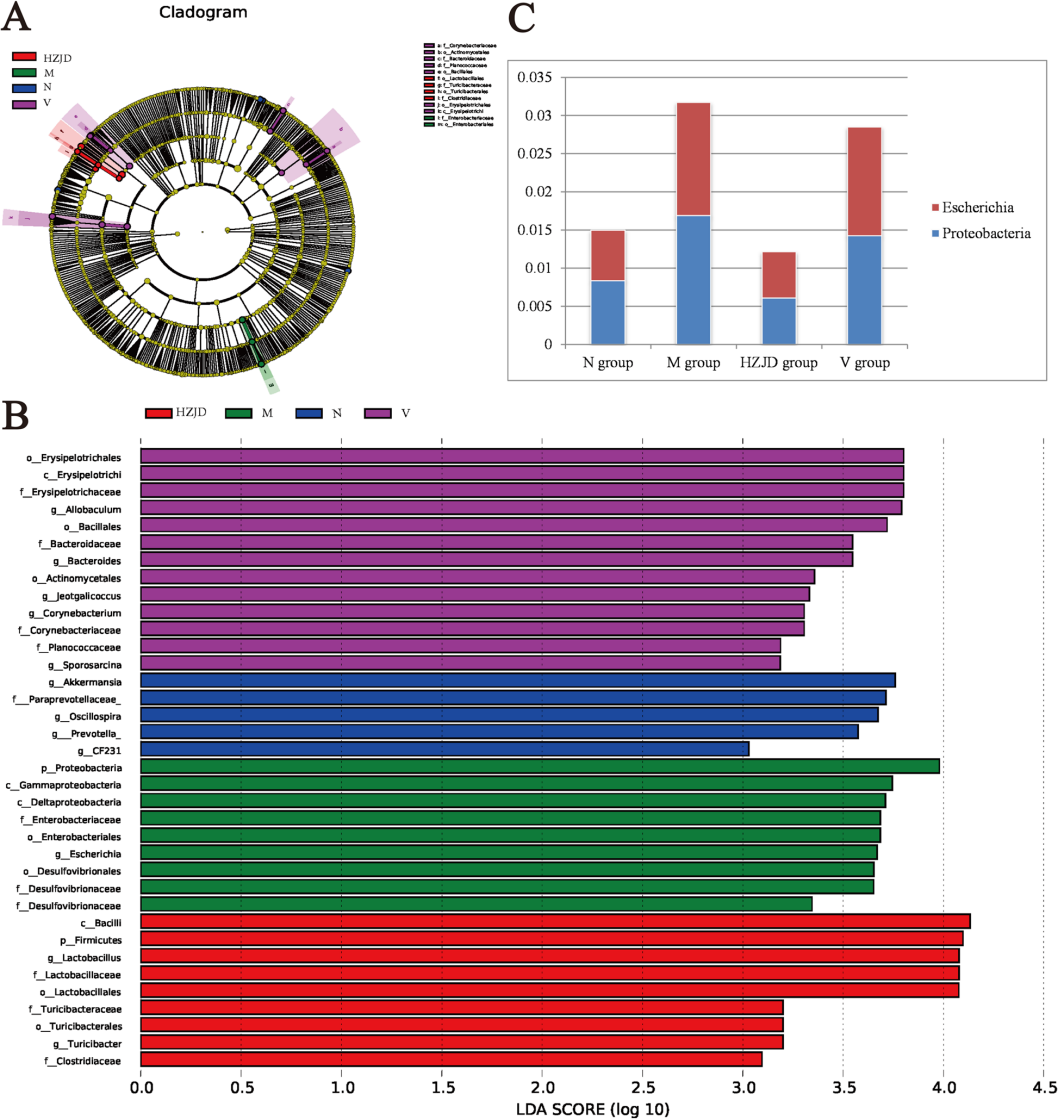
The potential biomarkers defined by LEfSe. **A** Cladogram. Each circle represents the classified level from phylum to species. The diameter of the small circle is proportional to the relative abundance of gut microbiota. **B** Histogram. The threshold on the logarithmic LDA score for discriminative features was set to 3.0. **C** Abundance of *Escherichia* and *Proteobacteria* in each group

We used the LEfSe method to identify the specific bacterial phylotypes that were differentially altered between the N, M, HZJD, and V groups. A cladogram was obtained from the LEfSe method (LDA > 3.0), and the results showed that the abundances of the genera *Akkermansia, Oscillospira, Prevotella,* and *CF231* were significantly higher in the N group. The phylum *Proteobacteria* and the genus *Escherichia* were significantly enriched in the M group (Fig. 8A and B). Following HZJD and vitacoenzyme treatment, the abundance of the phylum *Proteobacteria* and the genus *Escherichia* were decreased, and the therapeutic effect of HZJD was better than that of vitacoenzyme (Fig. 8C). The phylum *firmicutes* and the genera *Lactobacillus* and *Turicibacter* were significantly enriched in the HZJD group, and the genera *Allobaculum, Bacteroides, Jeotgalicoccus, Corynebacterium,* and *Sporosarcina* were significantly enriched in the V group (Fig. 8A and B).

### PCA KEGG function prediction

**Fig. 9:**
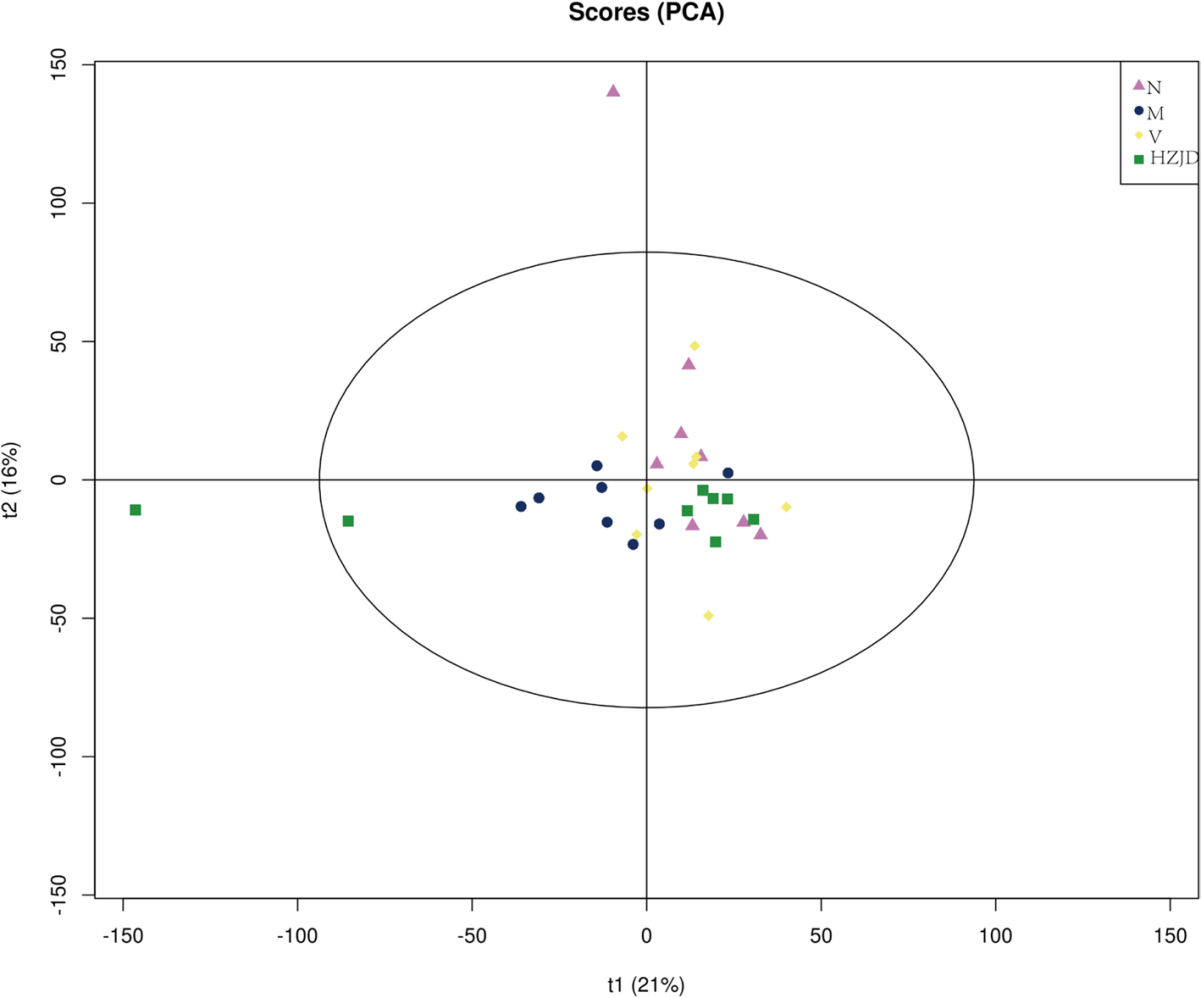
PCA KEGG function prediction result

KEGG function prediction analysis is an effective method to study the changes in metabolic function of community samples to adapt to environmental changes. PCA was thus used to explore the similarity or difference of the functions of different groups at an overall level. Fig. 9 shows that the samples of the N, HZJD, and V groups clustered together, the M group was separated from the N, HZJD, and V groups.

## Discussion

GC comprises the following precancerous steps: chronic active gastritis → chronic atrophic gastritis → gastric intestinal metaplasia → gastric epithelial dysplasia [18]. Inflammation, cell proliferation and apoptosis, autophagy dysfunction, energy metabolism disorders, and other factors may contribute to the development process, although the exact pathogenesis is still unclear [14, 17, 19, 20]. The common therapies used for CAG focus on symptomatic treatment and are often not completely effective. Strategies involving TCM are, however, becoming increasingly accepted. In TCM, turbid toxicity is an important cause of CAG, leading to disharmony between the spleen and the stomach and increases endogenous dampness and heat. The use of HZJD is based on turbid toxic syndrome. It functions to clear away heat, and detoxify and invigorate the spleen to remove dampness and has achieved good results in the treatment of CAG [14, 19]. However, the regulating effect of HZJD on the intestinal flora is still unclear. In this study, we used HPLC to control the quality of the HZJD decoction studied, H&E staining to evaluate the therapeutic effect of HZJD on CAG pathology, and 16S rRNA sequencing to evaluate the regulating effect of HZJD on the intestinal flora of CAG rats.

There is little doubt that the intestinal flora plays an important role in the development and progression of gastric diseases. Disturbances of the intestinal microbiome can induce inflammation, abnormal cell proliferation and apoptosis, and oxidative stress, which all may be associated with the development of CAG [21, 22]. A recent study has shown that there are significant differences in the microbiome between patients with GC, intestinal metaplasia, and chronic gastritis [23–25]. Thus, understanding what constitutes a health-promoting (eubiotic) or disease-promoting (dysbiotic) microbial community has become the focus of recent research [26].

The Venn diagram showed that 1,093 species were uniform between the four groups. These species may play an important role in the stability and function of a CAG rat intestinal microecological environment. However, 811 species were only found in the CAG model group, and these species may be related to the occurrence of CAG and are worthy of further exploration.

A rarefaction curve and species accumulation curve were generated and demonstrated the reliability of the sequencing data in this study. The Chao1 and ACE indices were significantly increased in the M group, indicating that the intestinal flora of CAG rats had a higher diversity. Prior reports have suggested that changes in microbial composition and diversity are essential to promote inflammation, proliferation, and tumor progression [27]. Moreover, our previous research demonstrated that abnormal cell proliferation and apoptosis mechanisms play an important role in the malignant transformation of the gastric mucosa [14, 28]. Therefore, we speculated that changes in the diversity of the intestinal flora in CAG may be an important factor for the occurrence of CAG. Following HZJD treatment, the Chao1 and ACE indices were significantly decreased, with no statistical significance compared to the N group, indicating that HZJD could effectively regulate the diversity of the CAG intestinal flora. PCA, NMDS and UPGMA results indicated that the intestinal flora microbial composition in CAG rats had changed, and the HZJD group exhibited a therapeutic effect on the community structure of the intestinal flora in CAG rats.

*Akkermansia* and *Oscillospira* are normal bacteria in the intestine and are crucial in maintaining the integrity and anti-inflammatory state of the gastrointestinal tract, while *Prevotella* is a dominant bacterial genus within the gut [29, 30]. The abundance of these bacteria were significantly enriched in the N group, which may be contribute to gastrointestinal health. The presence of *Proteobacteria* is a microbial symbol of intestinal flora imbalance, and an increase in *Proteobacteria* can cause inflammation of the gastrointestinal tract and destroy intestinal homeostasis [31]. *Escherichia* is one of the most common causes of several bacterial infections in humans and animals, is the prominent cause of gastroenteritis, and can induce apoptosis in gastric cells [32, 33]. In this study, the abundance of *Escherichia* and *Proteobacteria* increased significantly in the M group, which may be a cause of gastrointestinal inflammation and gastric mucosal atrophy. Following HZJD treatment, the abundance of the phylum *Proteobacteria* and the genus *Escherichia* decreased, indicating that HZJD could alleviate gastrointestinal inflammation by regulating the abundance of *Proteobacteria* and *Escherichia*. *Firmicutes* interact with endogenous beneficial microbes to restore intestinal homeostasis and are beneficial to a host’s intestinal environment [34]. *Lactobacillus* has been characterized as a type of probiotic in the digestive tract, and can maintain the microecological balance of the gastrointestinal tract. Moreover, dynamic monitoring of *Lactobacillus* levels has important clinical significance for predicting the development of GC [6]. *Lactobacillus* can also regulate the immune system, reducing the risk of allergies and cancer, and can reduce free radical oxidizing substances [35]. Studies have found that *Lactobacillus* can reduce the adhesion of *Helicobacter pylori* (H*p*), inhibit H*p* infection, and alleviate H*p*-related gastritis [36–38]. *Turicibacter* is an important member of the gut microbiota and is considered to be a healthy bacterial genera with anti-inflammatory effects [39–41]. CAG is a process of inflammatory cancer conversion, in which continuous inflammatory stimulation promotes the growth of GC cells and increases the chance of gene mutation. Imbalance in the intestinal flora can lead to the production of inflammatory factors and can cause gastric mucosal damage [42, 43]. In this study, the abundance of *Firmicutes, Lactobacillus*, and *Turicibacter* was significantly increased in the HZJD group, indicating that HZJD can increase the abundance of healthy bacterial genera, improving the anti-inflammatory ability of the gastrointestinal tract, and therefore protecting against the development of CAG. The genera *Allobaculum*, *Bacteroides*, *Jeotgalicoccus*, *Corynebacterium*, and *Sporosarcina* were significantly enriched in the V group. *Allobaculum* can produce short-chain fatty acids (SCFAs), and bacteria that produce SCFAs have previously been shown to protect mucous membranes from damage from pathogens by providing colonic cell nutrition and reducing inflammation, which are beneficial to the host [44]. However, conditional pathogenic bacteria, such as *Bacteroides* and *Sporosarcina*, were also found to be present in the V group.

Based on the KEGG database, PICRUSt software was used for function prediction. PCA result suggested that the M group had significant differences in the functions of the intestinal flora, while the N, HZJD, and V groups had similar enrichments of functions, which also proved the regulating effect of HZJD on the intestinal flora.

## Conclusion

In conclusion, these results indicated that HZJD exhibited a therapeutic effect on the diversity, community structure, and relative abundance of the intestinal flora in CAG rats, which may represent one of the mechanisms underlying the treatment effects of HZJD in CAG.

## Supplementary Information

**Additional file 1.**

## Data Availability

The datasets used and analyzed in the current study are available from the corresponding author upon reasonable request. All data generated or analyzed in this study are included in this article.

## Abbreviations

CAG: Chronic atrophic gastritis
GC: Gastric carcinoma
TCM: Traditional Chinese medicine
HZJD: Hua-Zhuo-Jie-Du
MNNG: 1-Methyl-3-nitro-1-nitrosoguanidine
V: Vitacoenzyme
M: Model
N: Normal
SD: Sprague–Dawley
HPLC: High-performance liquid chromatography
H&E: Hematoxylin–Eosin
KEGG: Kyoto encyclopedia of genes and genomes
PCA: Principal component analysis
LEfSe: LDA effect size
UPGMA: Unweighted pair group method with arithmetic mean
