# Supplementary material for: 16S rRNA sequencing-based evaluation of the protective effects of Hua-Zhuo-Jie-Du on rats with chronic atrophic gastritis
Source: BMC Complement Med Ther. 2022 Mar 16;22:71. doi: 10.1186/s12906-022-03542-z (PMC8928654; doi:10.1186/s12906-022-03542-z)
Supplement: Supplementary file 1 — Additional file 1. [file 12906_2022_3542_MOESM1_ESM.docx]

| **Sample** | **Raw PE** | **Clean PE** | **Base**  **(nt)** | **AvgLen**  **(nt)** | **Q20**  **(%)** | **Q30**  **(%)** | **GC**  **(%)** | **Effective**  **(%)** |
| --- | --- | --- | --- | --- | --- | --- | --- | --- |
| N 1 | 84,598 | 63,711 | 26,376,579 | 414 | 98.5 | 94.75 | 53.67 | 75.31 |
| N 2 | 86,804 | 61,255 | 25,390,766 | 415 | 98.52 | 94.97 | 53.65 | 70.57 |
| N 3 | 85,080 | 65,484 | 27,233,738 | 416 | 98.48 | 94.93 | 53.78 | 76.97 |
| N 4 | 81,224 | 68,200 | 28,619,698 | 420 | 98.45 | 94.79 | 53.1 | 83.97 |
| N 5 | 81,297 | 62,466 | 25,935,043 | 415 | 98.53 | 94.98 | 53.84 | 76.84 |
| N 6 | 91,443 | 66,773 | 27,830,562 | 417 | 98.46 | 94.8 | 53.47 | 73.02 |
| N 7 | 80,003 | 62,079 | 25,863,587 | 417 | 98.54 | 94.96 | 54.12 | 77.6 |
| N 8 | 99,560 | 62,428 | 26,039,416 | 417 | 98.27 | 94.27 | 53.33 | 62.7 |
| M 1 | 82,921 | 57,501 | 24,027,983 | 418 | 98.46 | 94.77 | 53.83 | 69.34 |
| M 2 | 86,429 | 61,422 | 25,585,431 | 417 | 98.43 | 94.74 | 54.13 | 71.07 |
| M 3 | 92,704 | 64,324 | 27,102,248 | 421 | 98.39 | 94.57 | 53.07 | 69.39 |
| M 4 | 89,218 | 60,381 | 25,242,425 | 418 | 98.43 | 94.75 | 53.98 | 67.68 |
| M 5 | 98,478 | 66,381 | 27,880,380 | 420 | 98.4 | 94.56 | 53.6 | 67.41 |
| M 6 | 83,950 | 56,821 | 23,850,280 | 420 | 98.35 | 94.47 | 53.48 | 67.68 |
| M 7 | 96,487 | 68,242 | 28,730,872 | 421 | 98.36 | 94.48 | 53.65 | 70.73 |
| M 8 | 88,107 | 66,121 | 27,527,010 | 416 | 98.45 | 94.79 | 53.73 | 75.05 |
| V 1 | 96,814 | 61,606 | 25,550,434 | 415 | 98.54 | 95.03 | 53.29 | 63.63 |
| V 2 | 92,383 | 60,022 | 25,040,262 | 417 | 98.59 | 95.09 | 53.82 | 64.97 |
| V 3 | 88,367 | 64,548 | 27,404,925 | 425 | 98.4 | 94.57 | 52.58 | 73.05 |
| V 4 | 94,525 | 64,908 | 26,913,395 | 415 | 98.44 | 94.74 | 52.84 | 68.67 |
| V 5 | 91,829 | 63,695 | 26,575,048 | 417 | 98.55 | 95.02 | 53.52 | 69.36 |
| V 6 | 92,513 | 62,333 | 25,854,939 | 415 | 98.54 | 95.05 | 53.6 | 67.38 |
| V 7 | 80,957 | 58,252 | 24,373,827 | 418 | 98.55 | 95.01 | 52.98 | 71.95 |
| V 8 | 94,017 | 64,232 | 26,875,849 | 418 | 98.47 | 94.85 | 53.77 | 68.32 |
| HZJD 1 | 98,595 | 63,076 | 26,341,599 | 418 | 98.51 | 94.91 | 53.35 | 63.97 |
| HZJD 2 | 94,164 | 67,356 | 28,088,539 | 417 | 98.44 | 94.75 | 53.24 | 71.53 |
| HZJD 3 | 91,624 | 63,879 | 26,614,120 | 417 | 98.52 | 94.99 | 53.34 | 69.72 |
| HZJD 4 | 81,678 | 61,539 | 25,474,445 | 414 | 98.57 | 95.06 | 53.2 | 75.34 |
| HZJD 5 | 93,274 | 68,687 | 28,611,174 | 417 | 98.61 | 95.21 | 53.88 | 73.64 |
| HZJD 6 | 93,710 | 66,026 | 27,647,964 | 419 | 98.44 | 94.77 | 53.86 | 70.46 |
| HZJD 7 | 92,820 | 60,285 | 25,309,221 | 420 | 98.4 | 94.66 | 53.29 | 64.95 |
| HZJD 8 | 96,218 | 66,850 | 28,138,619 | 421 | 98.39 | 94.57 | 53.02 | 69.48 |

**Supplementary material l: The effective sequence number and sequence length of the sample**
